# Detrended Fluctuation Analysis: A Scale-Free View on Neuronal Oscillations

**DOI:** 10.3389/fphys.2012.00450

**Published:** 2012-11-30

**Authors:** Richard Hardstone, Simon-Shlomo Poil, Giuseppina Schiavone, Rick Jansen, Vadim V. Nikulin, Huibert D. Mansvelder, Klaus Linkenkaer-Hansen

**Affiliations:** ^1^Department of Integrative Neurophysiology, Center for Neurogenomics and Cognitive Research, Neuroscience Campus Amsterdam, VU University AmsterdamAmsterdam, Netherlands; ^2^Department of Psychiatry, VU University Medical Center, Neuroscience Campus AmsterdamAmsterdam, Netherlands; ^3^Neurophysics Group, Department of Neurology, Charité – UniversitätsmedizinBerlin, Germany

**Keywords:** long-range temporal correlations, criticality, ongoing oscillations, detrended fluctuation analysis, scale-free dynamics

## Abstract

Recent years of research have shown that the complex temporal structure of ongoing oscillations is scale-free and characterized by long-range temporal correlations. Detrended fluctuation analysis (DFA) has proven particularly useful, revealing that genetic variation, normal development, or disease can lead to differences in the scale-free amplitude modulation of oscillations. Furthermore, amplitude dynamics is remarkably independent of the time-averaged oscillation power, indicating that the DFA provides unique insights into the functional organization of neuronal systems. To facilitate understanding and encourage wider use of scaling analysis of neuronal oscillations, we provide a pedagogical explanation of the DFA algorithm and its underlying theory. Practical advice on applying DFA to oscillations is supported by MATLAB scripts from the Neurophysiological Biomarker Toolbox (NBT) and links to the NBT tutorial website http://www.nbtwiki.net/. Finally, we provide a brief overview of insights derived from the application of DFA to ongoing oscillations in health and disease, and discuss the putative relevance of criticality for understanding the mechanism underlying scale-free modulation of oscillations.

## Introduction

When investigating nature we often discard the observed variation and describe its properties in terms of an average, such as the mean or median (Gilden, 2001). For some objects or processes, however, the average value is a poor description, because they do not have a typical or “characteristic” scale. Such systems are broadly referred to as “scale-free” (Bassingthwaighte et al., 1994). There is growing evidence that physiological processes can exhibit fluctuations without characteristic scales and that this scale-free dynamics is important for their function (Bassingthwaighte et al., 1994; Bak, 1996; Goldberger et al., 2002; Stam, 2005; Ghosh et al., 2008; He et al., 2010; West, 2010). Detrended fluctuation analysis (DFA; Peng et al., 1994), a method for analyzing scaling behavior in time series, has played a critical role in this success. We believe, however, that DFA could prove valuable to a wider community of neuroscientists than its current users. Thus, the aim of this paper is to promote and facilitate investigations of the scale-free amplitude modulation of ongoing neuronal oscillations with the use of DFA (Linkenkaer-Hansen et al., 2001).

Our paper is structured as follows. First, we provide a beginner’s introduction to the Section “Fundamental Concepts Required to Understand DFA.” This is followed by the presentation of “The DFA” and the special requirements regarding “DFA applied to neuronal oscillations.” With the theory covered, the reader is referred to MATLAB code and tutorials in the Section “Try it Yourself Using the Neurophysiological Biomarker Toolbox (NBT).” Finally, we illustrate the value of DFA in “Insights from the application of DFA to neuronal oscillations.”

## Fundamental Concepts Required to Understand DFA

To understand how the DFA algorithm quantifies some of the properties of scale-free fluctuations, we introduce the concepts of self-affinity and stationarity and show how they apply to scale-free signals.

### Self-affinity

Self-affinity is a property of fractal time series (Mandelbrot, 1967; Turcotte, 1997). It is a special case of self-similarity, according to which a small part of a fractal structure is similar to the whole structure. When this small part is an exact replica of the whole then the fractal is exact, which is the case for purely mathematical and geometrical fractals (e.g., the van Koch curve and the Mandelbrot tree; Peitgen et al., 1992). When the self-similarity is expressed in terms of statistical properties (e.g., the mean and standard deviation for a portion of a fractal are scaled versions of the mean and standard deviation of the whole) then the fractal is a statistical fractal. Whilst the self-similarity property is isotropic and applies along all the dimensions of a fractal object, self-affinity describes anisotropic scaling where statistical properties of the fractal scale differently along different dimensions. In the case of a time series, the time dimension is rescaled.

**Figure 1:**
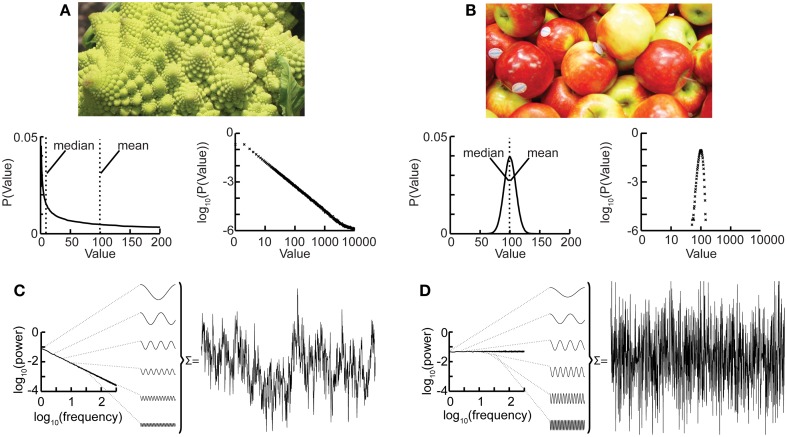
**The Roman cauliflower is a striking example of self-similarity in nature**. **(A)** The cauliflower is composed of flowers that are similar to the entire cauliflower. These smaller flowers, in turn, are composed of flowers that are similar to the smaller flowers. The self-similarity is apparent on at least four levels of magnification, thereby illustrating the scale-free property that is a consequence of self-similarity (*bottom left*). A hypothetical distribution of the likelihood of flowers on a cauliflower having a certain size. This property is captured by the power-law function. The mean or median of a power-law, however, provide a poor representation of the scale-free distribution (and in a mathematical sense is not defined) (*bottom right*). The power-law function makes a straight line in double-logarithmic coordinates. The slope of this line is the exponent of the power-law, which captures an important property of scale-free systems, namely the relationship between the size of objects or fluctuations on different scales. **(B)** As the size of apples shows smaller variation they are well described by taking an average value such as the mean or median. (*bottom left*) Hypothetical distribution showing the likelihood of apples having a certain size. Both the mean and median are good statistics to convey the size of the apples. (*bottom right*) Plotting the normal distribution on double-logarithmic coordinates has little effect on the appearance of the distribution, which still shows a characteristic scale. **(C)** Time-signals can also be viewed as self-affine as they can be transformed into a set of sine-waves of different frequencies. In a 1/*f* signal the lower frequency objects have larger amplitude than the higher frequency objects which we can compare with there being fewer large cauliflowers than there are small cauliflowers. **(D)** A white-noise signal is also self-affine, but now the lower frequency objects have the same amplitude as the higher frequency objects meaning that only the high-frequency fluctuations are visible in the signal.

Nature hosts some intriguing examples of self-similar structures, such as the Roman cauliflower (*Romanesco broccoli*), in which almost exact copies of the entire flower may be recognized on multiple smaller scales (Figure 1A). Physiological time series may exhibit statistical self-affine properties (Eke et al., 2000, 2002). Self-affine processes and self-similar structures have in common that the statistical distribution of the measured quantity follows a power-law function, which is the only mathematical function without a characteristic scale. Self-affine and self-similar phenomena are therefore called “scale-free.”

Considering again the example of the *Romanesco broccoli*, we can say that it is a “scale-free” structure, because there is no typical size of flower on the cauliflower, with the frequency of a certain size of flower being inversely proportional to its size. A scale-free time series will in a similar fashion be composed of sine-waves with amplitudes inversely proportional to their frequency (Figure 1C), seen as a straight line when the power spectrum is plotted on double-logarithmic axis. This is in contrast to the wide variety of objects that have a typical scale, e.g., the size of the apples on a tree. None of them will be very small or very large; rather, they will form a Gaussian distribution centered on some characteristic size, which is well represented by the mean of the distribution. Qualitatively, the characteristic scale is present at the expense of rich variability. Similarly, a time series in which all frequencies are represented with the same amplitude will lack the rich variability of the scale-free time series and is referred to as “*white-noise*” (Figure 1D). Whereas phenomena with characteristic scales are well defined by their mean and standard deviation (Figures 1B,D), scale-free phenomena are better described by the exponent of a power-law function, because it captures the relationship between objects or fluctuations on different scales (Figures 1A,C).

Let us now introduce the mathematical definitions:

A non-stationary stochastic process is said to be *self-affine* in a statistical sense, if a rescaled version of a small part of its time series has the same statistical distribution as the larger part. For practical purposes, it is sufficient to assess the standard deviation. Thus, the process, *Y*, is self-affine if for all windows of length *t*:

(1) $$Y\left(Lt\right)\equiv L^{H}Y\left(t\right)$$

where:

- “*Y*(*Lt*)” and “*Y*(*t*)” are values of a process at time windows of length *Lt* and *t*, respectively.
- “*L*”: window length factor
- “*H*”: Hurst parameter, dimensionless estimator of self-affinity
- “≡”: the standard deviation on both sides of the equation are identical (Beran, 1994).

To illustrate the implications of this definition for the property of a self-affine process, we consider a self-affinity parameter of 0.75 and derive the standard deviation for two and three times the length of the time-scale. To double the time-scale, we set *L* = 2;

$$\begin{array}{llll} & Y\left(2t\right)\equiv 2^{0.75}Y\left(t\right) & & \\ & Y\left(2t\right)\equiv 1.68Y\left(t\right) & & \end{array}$$

Therefore, the standard deviation of a signal twice the length of *y*(*t*) is 1.68 times larger than that of the original signal *y*(*t*).

Tripling the window size with *L* = 3 gives;

$$\begin{array}{llll} & Y\left(3t\right)\equiv 3^{0.75}Y\left(t\right) & & \\ & Y\left(3t\right)\equiv 2.28Y\left(t\right) & & \end{array}$$

The standard deviation increases by a factor of 2.28. In other words, with a self-affinity parameter *H* = 0.75, the standard deviation grows with increasing window size according to the power-law, *L^H^*. This mathematical formulation shows another property of self-affine processes which is scale-invariance: the scaling of the standard deviation is not dependent on the absolute scale. A signal exhibiting the described behavior is also said to exhibit “scale-free” fluctuations with a “power-law scaling exponent” *H*. *H* is the Hurst-coefficient (Mandelbrot and Wallis, 1969) and ranges between 0 and 1. *H* approaching 1 describes a signal of smooth appearance, typically meaning that high values are followed by high values (i.e., there are dependencies over time), while H close to 0 is a signal with rough, “hairy” appearance, which typically means faster switching between high and low values.

The estimation of the scaling exponent is particularly interesting for neuronal oscillation dynamics, because it can reveal the presence of long-range temporal correlations (LRTC) in neuronal network oscillations (Linkenkaer-Hansen et al., 2001). In the following sections we will show you how.

### Stationary and non-stationary processes

Definition: a process *X*(*t*) is stationary if the distribution of *X*(*t*) is independent of t, the joint distribution of *X*(*t*_1_ + τ) and *X*(*t*_2_ + τ) is independent of τ and similarly – for all *k* – for the joint distributions of *X*(*t*_1_ + τ) … *X*(*t_k_* + τ) (Mandelbrot, 1982).

When performing scale-free analysis of a time series, it is essential to have a model of whether the underlying process is stationary. This is because many of the methods used on a time series to estimate *H* make assumptions about whether the process is stationary or not. For example, self-affinity as described above only applies to non-stationary processes, because by definition the variance of a stationary process does not alter with the amount of time looked at (Beran, 1994).

Scale-free processes which are stationary are usually modeled as fractional Gaussian noise (fGn), and non-stationary processes are modeled as fractional Brownian motion (fBm). Nevertheless, there is a strong relationship between these two types of processes in that, by definition, the increments of a fBm process are modeled as a fGn process with the same Hurst parameter, for more details on these models (see Mandelbrot, 1982; Eke et al., 2000). This relationship allows us to apply the definition of self-affinity given above to a stationary fGn process, by first converting it into its non-stationary fBm equivalent as follows. Given the time series *y*(*t*), we define the *signal profile* as the cumulative sum of the signal:

(2) $$x\left(t\right)=\overset{t}{\underset{k=1}{\sum }}y\left(k\right)-\left\langle y\right\rangle$$

where (*y*) is the mean of the time series. The subtraction of the mean eliminates the global trend of the signal. The advantage of applying scaling analysis to the signal profile instead of the signal, is that it makes no *a priori* assumptions about the stationarity of the signal. When computing the scaling of the signal profile, the resulting scaling exponent, α, is an estimation of *H*. If α is between 0 and 1, then *x* was produced by a stationary process which can be modeled as a fGn process with *H* = α. If α is between 1 and 2 then *x* was produced by a non-stationary process, and *H* = α − 1 (Eke et al., 2000).

### Scaling of an uncorrelated stationary process

**Figure 2:**
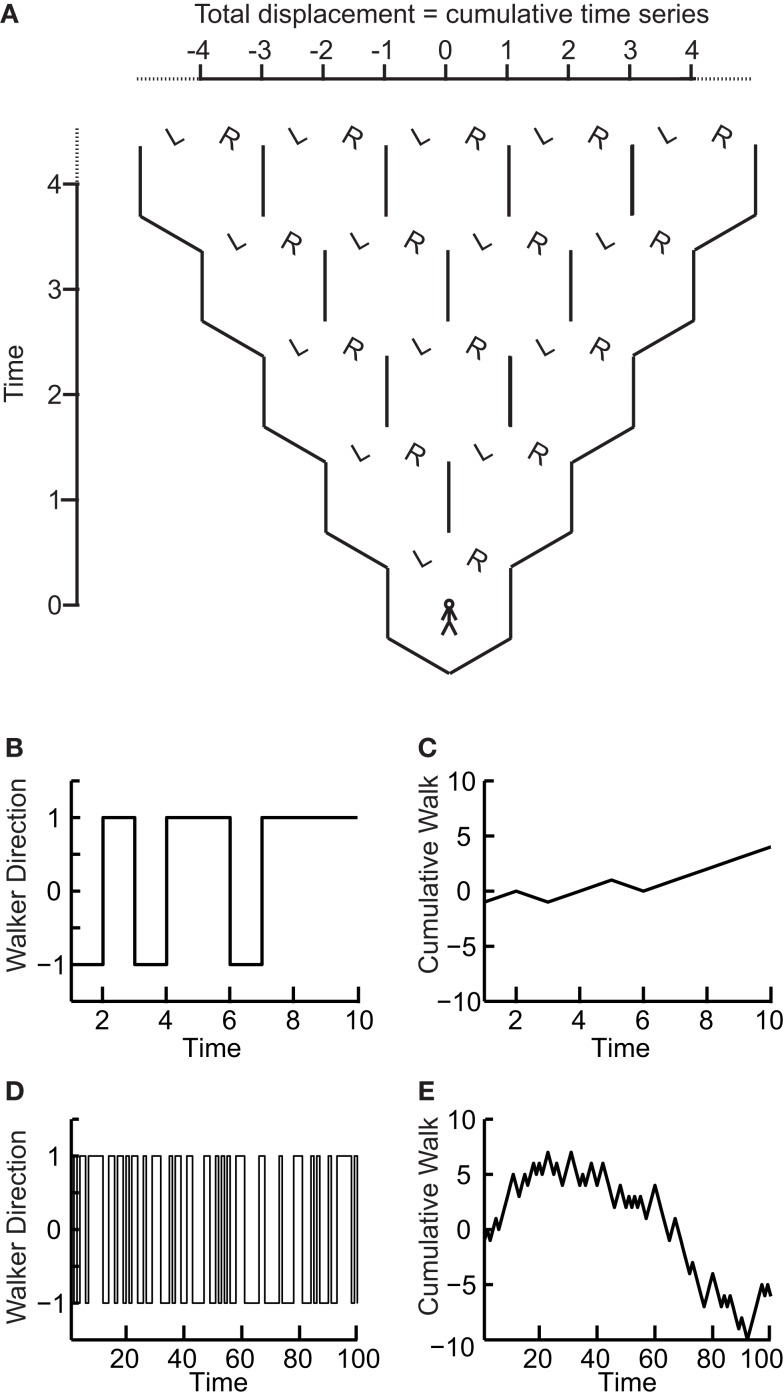
**The “random walk”: the signal profile of a stationary time series may reveal self-affinity**. **(A)** At each time step a walker moves randomly to the left (−1) or right (+1) with equal probability. At any time step the probability of being at a certain displacement from the origin depends on the number of different paths that could take the walker there. **(B)** The walker’s steps form a time series that is stationary as its value does not depend on time. **(C)** The signal profile can take arbitrarily large values as the time increases. **(D)** Looking at the walker time series on a longer time-scale the standard deviation does not change as the signal cannot take larger values. **(E)** The cumulative sum, or random walk process, on a longer time-scale shows larger variance than on the shorter time-scale **(C)** therefore the walker may exhibit self-affinity or scale-free behavior.

We now show that the scaling of a so-called random walk process can be used to infer whether a time series is uncorrelated. A random walk is a non-stationary probabilistic process derived from the cumulative sum of independent random variables, where each variable has equal probability to take a value of 1 or −1. Imagine a walker that at each time step can either take one step left (−1) or right (+1) with equal probabilities (Figure 2A). The sequence of the steps representing independent random variables forms a stationary time series as it can only take two values which do not depend on time (Figures 2B,D). If we calculate the standard deviation of this time series for differently sized time windows we will not see a scaling effect as there will always on average be an equal number of 1’s and −1’s. As the probability of taking either action does not depend on any previous actions, the process is said to be “*memory-less*.”

Now, if we compute the cumulative sum of this time series, using Eq. 2 for obtaining the random walk, we can calculate the distance that the walker deviates from the zero line where it started (following a given number of steps; Figures 2A,C,E). This distance changes with the number of steps that the walker has taken. Therefore, it is possible to calculate how the standard deviation of distance from the origin (referred to as random walk fluctuations) changes depending on the number of steps that the walker has taken.

We can calculate this by working out the relationship between the displacement, *x*, at time *t* and time *t* + 1. If at time *t* the walker is at position *x_t_* then at time *t* + 1 the walker will be at position *x_t_* − 1 or *x_t_* + 1 with equal likelihood. Therefore, we can calculate the mean square displacement at time *t* + 1:

(3) $$\begin{array}{l} \left\langle x_{t+1}^{2}\right\rangle =\frac{\left\langle {\left(x_{t}+1\right)}^{2}+{\left(x_{t}-1\right)}^{2}\right\rangle }{2}\\ =\frac{\left\langle x_{t}^{2}+2x_{t}+1+x_{t}^{2}-2x_{t}+1\right\rangle }{2}\\ \left\langle x_{t+1}^{2}\right\rangle =\frac{2\left\langle x_{t}^{2}\right\rangle +2}{2}=\left\langle x_{t}^{2}\right\rangle +1 \end{array}$$

Let us define the starting position to be 0, i.e., the mean square displacement at time 0 is:

$$\left\langle x_{0}^{2}\right\rangle =0$$

Now, we can calculate the mean square displacement after an arbitrary number of steps by applying Eq. 3 iteratively:

$$\begin{array}{l} \left\langle x_{1}^{2}\right\rangle =\left\langle x_{0}^{2}\right\rangle +1=0+1=1\\ \left\langle x_{2}^{2}\right\rangle =\left\langle x_{1}^{2}\right\rangle +1=1+1=2\\ \left\langle x_{3}^{2}\right\rangle =\left\langle x_{2}^{2}\right\rangle +1=2+1=3\\ \ldots \\ \left(x_{L}^{2}\right)=L \end{array}$$

Thus, the mean square displacement after a walk of length *L* steps, or equivalently, the root-mean-square displacement after *L* steps is the square root of *L*:

(4) $${\left(\left\langle x_{L}^{2}\right\rangle \right)}^{0.5}=L^{0.5}$$

For a zero mean signal, *x*, the root-mean-square displacement is the standard deviation. Thus, the cumulative sum of a randomly fluctuating zero mean signal will have the standard deviation growing with window length, *L*, according to a power-law with the exponent of 0.5. Now, recall from Eq. 1 that if the standard deviation of a signal scales by a factor *L^H^* according to the length of the signal, *L*, then the process exhibits self-affinity with Hurst exponent *H*. Thus, we have derived that a stationary randomly fluctuating process has a signal profile, which is self-affine with a scaling exponent α = 0.5.

### Scaling of correlated and anti-correlated signals

**Figure 3:**
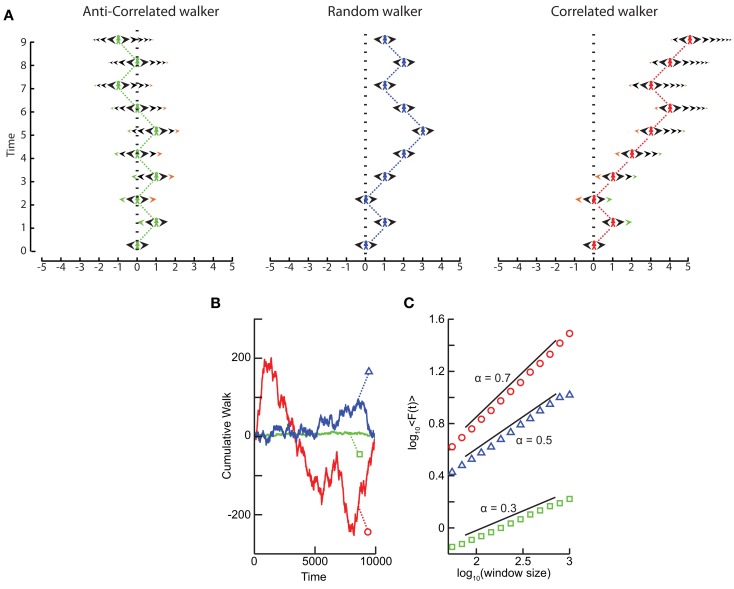
**Processes with a memory produce qualitatively, and quantitatively, different fluctuations compared to a random walk process**. **(A)** Correlations occur when the “walker’s” decision to follow a certain direction is influenced by its past actions. (Left) Path of an anti-correlated walker shown over time. At each time step the walker makes a decision based on a weighted choice between left and right. The weighted choice can be seen by the sum of the areas of the arrows pointing left and right. Each action the walker takes continues to influence future actions, with the walker being more likely to take the opposite action. This is illustrated as a gradual accumulation of arrows that refer to past actions, but also decrease in size over time, because the bias contributions of those actions decay over time. The green arrows show how the first action the walker takes (going Right) persists over time, with the influence getting smaller as time goes on seen by the green arrow size decreasing. (Center) Path of a random walker shown over time. The random walker is not influenced by previous actions and so always has equal probability of going left or right. (Right) Path of a correlated walker shown over time. Here each action the walker takes influences future actions by making the walker more likely to take that action. The green arrows show that by taking the action of going right at time 0, the walker is more likely to go right in future time steps with the influence getting smaller as time goes on. **(B)** Cumulative signal for a positively correlated process (*red*, *circle*) shows larger fluctuations over time than a random walker (*blue*, *triangle*). An anti-correlated signal (*green, square*) shows smaller fluctuations over time. **(C)** By looking at the average fluctuations for these different processes at different time-scales, we can quantify this difference. A random walker shows a scaling exponent of 0.5, with the positively correlated process having a larger exponent, and the anti-correlated process having a smaller exponent.

What happens to the self-affinity of a process when we add memory in the sense that the probability of an action depends on the previous actions that the walker has made? Different classes of processes with memory exist. Let us focus on those with positive correlations and those with anti-correlations. Anti-correlations can be seen as a stabilizing mechanism: any action the walker makes means that when taking future actions the walker will be more likely to take the opposite action (Figure 3A). This leads to smaller fluctuations on longer time-scales than seen by chance (Figure 3B). Positive correlations have the opposite effect: any action the walker takes makes it more likely to take that action in the future (Figure 3A). This leads to large fluctuations in the integrated signal (Figure 3B). We define a fluctuation function as the standard deviation of the signal profile:

(5) $$f\left(L\right)={\left(\left\langle x_{L}^{2}\right\rangle \right)}^{0.5}=L^{\alpha }$$

We note from Eq. (4) that this function grows as a power-law with self-affinity parameter α = 0.5 for a stationary random signal. Using Eq. (5) – and as shown in Figure 3C – it follows that if the fluctuations scale according to time with:

- 0 < α < 0.5 then the process has a memory, and it exhibits anti-correlations. (can be modeled by a fGn with *H* = α)
- 0.5 < α < 1 then the process has a memory, and it exhibits positive correlations. (can be modeled by a fGn with *H* = α)
- α = 0.5 then the process is indistinguishable from a random process with no memory. (can be modeled by a fGn with *H* = α)
- 1 < α < 2 then the process is non-stationary. (can be modeled as a fBm with *H* = α − 1).

For short-range correlations the scaling exponent will deviate from 0.5 only for short window sizes, because the standard deviation of the integrated signal in long windows will be dominated by fluctuations that have no dependence on each other. Thus, it is important to report the range where the scaling is observed. We return to the practical issues of identifying the scaling range in the section on “Insights from the application of DFA to neuronal oscillations.”

### Effects of trends on scaling

We have seen that calculating the fluctuation of signal profiles in windows of different sizes can be used to quantify the scale-free nature of time series. However calculating the fluctuations at a certain time-scale is strongly influenced by whether the signal has a steady trend on longer time-scales. This trend is unlikely to be part of a process on the time-scale of that window and may be removed by subtracting the linear trend in the window, and then calculating the standard deviation. This way we know that processes on scales larger than the given window size will only marginally influence the fluctuation function, Eq. (5).

**Figure 4:**
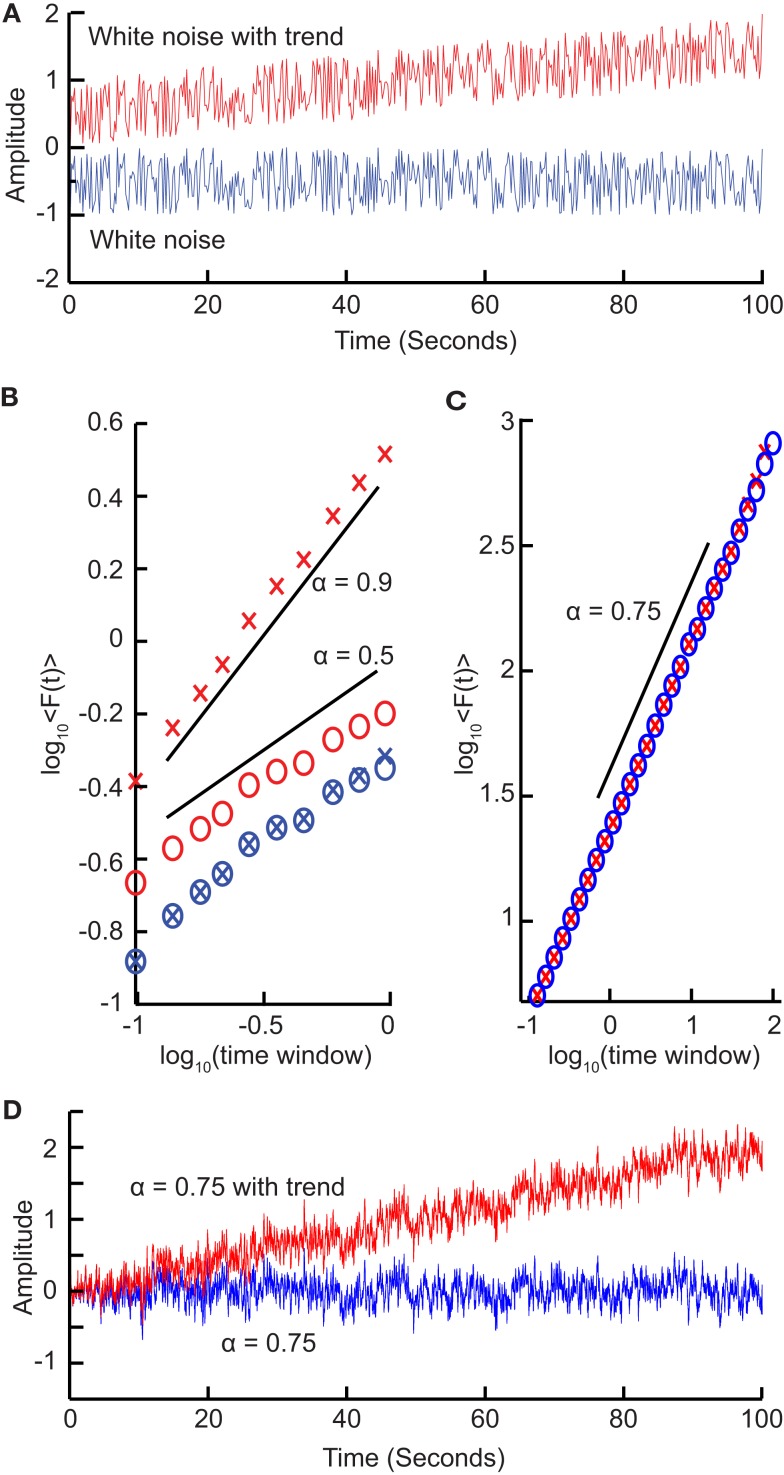
**Trends on longer time-scales can introduce false correlations into the signal**. **(A)** For a signal with a trend, the standard deviation will be larger (σ = 0.41) than the same signal with no trend (σ = 0.29). **(B)** Average fluctuations for a window size shown for a white-noise signal (*blue crosses*) and the same signal with a trend added (*red crosses*) show different scaling. By removing the linear trend of the integrated signal from each window before calculating the standard deviation (*circles*), we recover the scaling seen without the long-time-scale trend. **(C)** Importantly, detrending self-similar signals with trends (*red crosses*) also recovers the scaling of the original signal (*blue circles*). **(D)** Self-similar signal (α = 0.75) with trend (*red*) and without trend (*blue*) used in **(C)**.

To illustrate this, consider a white-noise signal with and without a slow trend (Figure 4A). The standard deviation of the integrated signal with a trend necessarily will be larger for any window size and, importantly, also grow faster with increasing window sizes compared to the signal without a trend (Figure 4B). Detrending the signal profile, however, efficiently reveals the true scaling of the signal with a superimposed trend both for uncorrelated (Figure 4B) and correlated (Figures 4C,D) signals. This is the basis for the robust performance of the DFA algorithm which we describe in the next section.

## The Detrended Fluctuation Analysis

**Figure 5:**
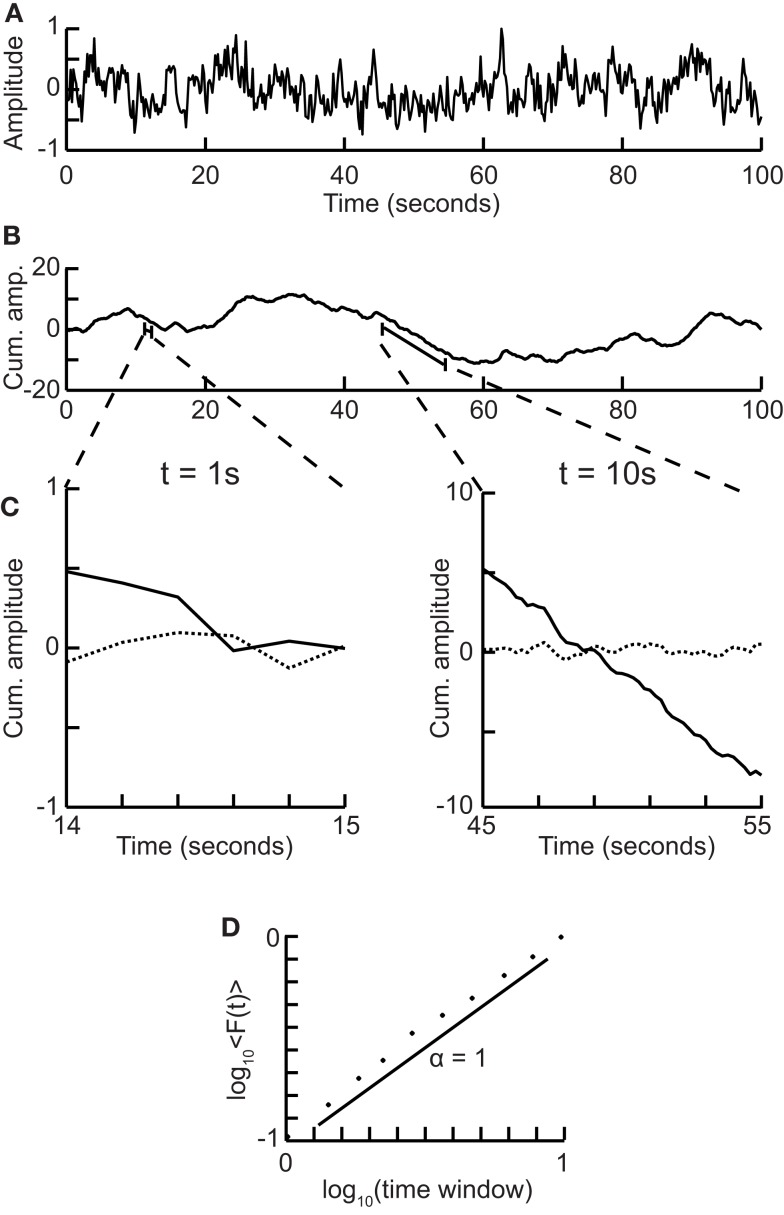
**Step-wise explanation of Detrended Fluctuation Analysis**. **(A)** Original time series. Taken from a 1/*f* signal sampled at 5 Hz with a duration of 100 s. **(B)** Cumulative sum of original signal shows large fluctuations away from the mean. **(C)** For each window size looked at, remove the linear trend from the signal, and then calculate the fluctuation. Two example window sizes shown with signal shown as solid line, and detrended signal shown as dotted line. **(D)** Plot the mean fluctuation per window size against window size on logarithmic axes. The DFA exponent is the slope of the best-fit line (α = 1).

Detrended fluctuation analysis, was introduced by Peng et al. (1994) to quantify LRTC with less strict assumptions about the stationarity of the signal than the auto-correlation function. This was supported with a set of online tutorials and datasets^1^ to allow researchers to investigate the method on real-life data (Goldberger et al., 2000). Since then, the algorithm has found widespread application as indicated by more than 1800 citations to (Peng et al., 1994; Google Scholar, September 2012), and it is one of the most commonly used methods to quantify the scale-free nature of physiological time series and their alteration in disease (Peng et al., 1995; Castiglioni et al., 2010; Frey et al., 2011). The DFA is based on the rationale described in the sections presented so far, and can be summarized as follows:

1. Compute the cumulative sum of the time series (Figure 5A) to create the signal profile (Figure 5B).
2. Define a set of window sizes, ***T***, which are equally spaced on a logarithmic scale between the lower bound of four samples (Peng et al., 1994) and the length of the signal.
  a. For each window length *t∈****T***
  a.i. Split the signal profile into a set (***W***) of separate time series of length *t*, which have 50% overlap.
  a.ii. For each window *w* **∈** ***W***
  a.ii.1. Remove the linear trend (using a least-squares fit) from the time series to create *w*^detrend^ (Figure 5C)
  a.ii.2. Calculate the standard deviation of the detrended signal, σ(w^detrend^)
  a.iii. Compute fluctuation function as the mean standard deviation of all identically sized windows:
  $$<F\left(t\right)>=\text{mean}\left(\sigma \left(\text{W}\right)\right)$$
3. Plot the fluctuation function for all window sizes, ***T***, on logarithmic axes (Figure 5D).
4. The DFA exponent, α, is the slope of the trend line in the range of time-scales of interest and can be estimated using linear regression (Figure 5D).

Here, we have chosen logarithmically spaced window sizes, because it gives equal weight to all time-scales when we fit a line in log-log coordinates using linear regression. The lower end of the fitting range is at least four samples, because linear detrending will perform poorly with less points (Peng et al., 1994). For the high end of the fitting range, DFA estimates for window sizes >10% of the signal length are more noisy due to a low number of windows available for averaging (i.e., less than 10 windows). Finally, the 50% overlap between windows is commonly used to increase the number of windows, which can provide a more accurate estimate of the fluctuation function especially for the long-time-scale windows.

The DFA exponent is interpreted as an estimation of the Hurst parameter, as explained with the random walker example, i.e., the time series is uncorrelated if α = 0.5. If 0.5 < α < 1 then there are positive correlations present in the time series as you are getting larger fluctuations on longer time-scales than expected by chance. If α < 0.5 then the time series is anti-correlated, which means that fluctuations are smaller in larger time windows than expected by chance.

Since DFA was first introduced several papers have tested the performance of DFA in relation to trends (Hu et al., 2001), non-stationarities (Chen et al., 2002), pre-processing such as artifact rejection (Chen et al., 2002), and coarse-graining (Xu et al., 2011). Other trend-removal techniques have been proposed, such as higher-order polynomial (Kantelhardt et al., 2001) or adaptive detrending (Riley et al., 2012); however, these have not yet been tested in the DFA analysis of neuronal oscillations.

## DFA Applied to Neuronal Oscillations

**Figure 6:**
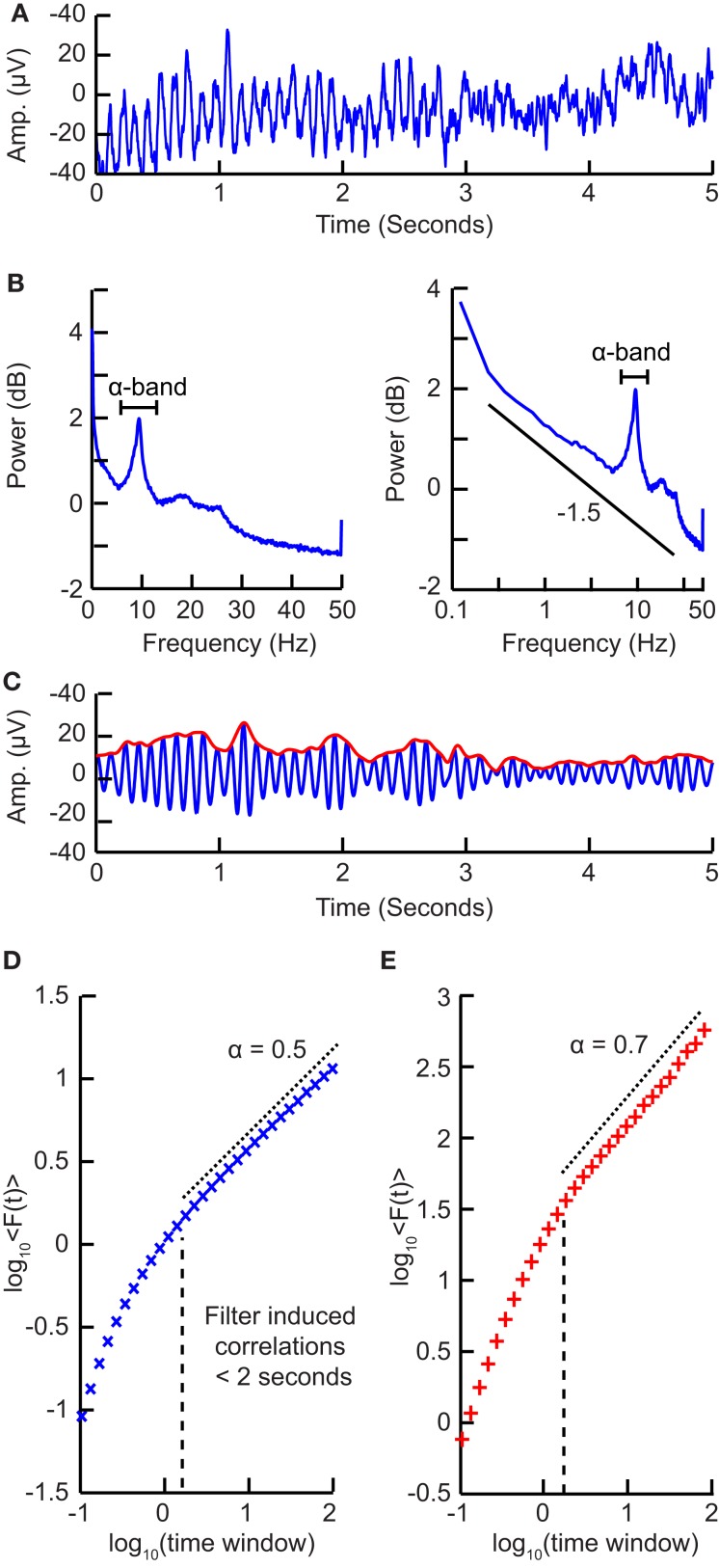
**Step-wise explanation of applying DFA to neuronal oscillations**. **(A)** EEG recording from electrode Oz shows clear oscillations during a 15 min eyes-closed rest session. Data were recorded at 250 Hz and band-passed filtered between 0.1 and 200 Hz. **(B)** Power spectrum (Welch method, zero padded) shown in logarithmic (left) and double-logarithmic axes (right), shows clear peak in the alpha band. **(C)** Signal in **(B)** filtered in the alpha band (8–13 Hz) using a fir filter with an order corresponding to the length of two 8 Hz cycles (*blue*). Amplitude envelope (*red*) calculated using the Hilbert transform. **(D)** DFA applied to the amplitude envelope of white-noise signal filtered using the same filter as in **(C)**. At time windows <2 s, filter-induced correlations are visible through a bend away from the 0.5 slope. **(E)** DFA applied to the amplitude envelope of the alpha band filtered EEG signal shows long-range temporal correlations between 2 and 90 s with exponent α = 0.71.

Synchronized activity between groups of neurons occurs in a range of frequencies spanning at least four orders of magnitude from 0.01 to 100 Hz (Buzsáki, 2006). The power spectral density plotted on double-logarithmic axes roughly follows a power-law distribution, but there are also several “peaks” seen along it, corresponding to the classical frequency bands (e.g., theta, alpha, beta, etc.; Figure 6B). In this section, we describe how to apply DFA to the amplitude modulation in these frequency bands, and show how they have been utilized in quantifying healthy and pathological conditions. We cannot apply DFA directly to the band-pass filtered signal, because it will appear as a strongly anti-correlated signal because of the peaks and troughs averaging out when computing the cumulative sum. Instead, we focus on the amplitude envelope of oscillations.

Our method consists of four steps:

1. Pre-processing of signals.
2. Create band-pass filter for the frequency band of interest.
3. Extract the amplitude envelope and perform DFA.
4. Determine the temporal integration effect of the filter to choose the window sizes for calculating the DFA exponent.

### Pre-processing of signals

Sharp transient artifacts are common in EEG signals. These large jumps in the EEG signal on multiple channels are, e.g., caused by electrode movement. Leaving these in the signal is likely to affect the DFA estimates, whereas removing them has little effect on the estimated exponent (Chen et al., 2002). Other artifacts from, e.g., eye movement, respiration heartbeat, sweat are also likely to disturb the estimate, thus they should be removed.

Another factor that can influence the DFA estimate is the signal-to-noise ratio of the signal. The lower this ratio, the more biased the estimated scaling is toward an uncorrelated signal. Simulations indicated that a SNR >2 is sufficient to accurately determine LRTC (Linkenkaer-Hansen et al., 2007).

### Filter design

To filter the EEG/MEG data (Figure 6A) we use a band-pass finite-impulse-response filter (FIR). This is used instead of an infinite impulse response filter (IIR) to avoid introducing long-range correlations in the signal before calculating the fluctuation function. The filter order for the FIR filter is recommended to be set to two cycles of the lowest frequency in order to accurately detect the oscillations while also limiting the temporal integration caused by the filter. In (Figure 6B) we can see a clear peak in the alpha band frequency range (8–13 Hz) and therefore we would band-pass filter in this frequency range with a filter order set to two cycles of 8 Hz.

### Extract the amplitude envelope and perform DFA

When applying DFA to neuronal oscillations, we are interested in how the amplitude of an oscillation changes over time. To calculate this we extract the amplitude envelope from the filtered signal by taking the absolute value of the Hilbert transform (Figure 6C; Nikulin and Brismar, 2005). The Hilbert transform is easily accessible in most programming languages (e.g., scipy.signal.Hilbert in Python (Scipy), Hilbert in Matlab). Wavelet transforms, however, have also been used to extract the amplitude envelope (Linkenkaer-Hansen et al., 2001). Once you have the amplitude envelope you can perform DFA on it. However, to decide which window sizes to calculate the exponent from, you first need to follow step 4.

### Determining the temporal integration effect of the filter

Filtering introduces correlation in the signal between the neighboring samples (e.g., due to the convolution in case of FIR filtering). Thus, including very small window sizes in the fitting range of the fluctuation function will lead to an overestimation of temporal correlations (Figure 6D). The effect of a specific filter on the DFA may be estimated using white-noise signals (where a DFA exponent of 0.5 is expected; Nikulin and Brismar, 2004):

a) Create 1000 white-noise signals each one corresponding to ∼1000 s.
b) Filter each signal using the filter designed in step 2.
c) Extract the amplitude envelopes of the filtered noise signals (step 3).
d) Perform DFA on each signal, and average all fluctuation functions.
e) Estimate the lowest fitting time window where the fluctuation function starts to curve away from an exponent of 0.5.

Now that you have the window sizes that have only negligible filter effect, you are finally able to calculate the DFA exponent (Figure 6E).

## Try it Yourself Using the Neurophysiological Biomarker Toolbox

The NBT was created to facilitate integration of multiple biomarkers and to support large-scale biomarker research in the Matlab environment. DFA has been implemented as part of the NBT. You can download NBT from http://www.nbtwiki.net, where you can also find further tutorials on using this toolbox. NBT can import various data formats (e.g., raw, .dat, .mat, .txt) into the NBT format. The NBT format is defined by three main .mat files: the first contains the signal stored in a matrix, the second contains information about the signal, the third contains the biomarker objects and it is automatically created when you compute a biomarker. The three files are named according to the NBT convention:

- *projectID.subjectID.date.condition.mat* for the signal
- *projectID.subjectID.date.condition_info.mat* for the signal information
- *projectID.subjectID.date.condition_analysis.mat* for the biomarkers.

After you have imported your data into NBT format a variety of actions can be performed on the data, from viewing and pre-processing data to biomarker computation, statistical analysis, and visualization. In the following, we show how a single biomarker, the DFA exponent, can be calculated using the MATLAB command line or a script.

You can also find this tutorial (with more details) online: http://www.nbtwiki.net/doku.php?id=tutorial:detrended_fluctuation_analysis_dfa

### Removing artifacts

Before performing any analysis you need to load the signal (already converted into NBT format) into the workspace. Type the following line in the command window to load the signal:


[Signal,SignalInfo,path]=nbt_load_file;


*Signal* and *SignalInfo* are the main variables on which NBT works, containing the signal and signal information respectively. Most of the NBT functions have these two variables as input and produce an updated version of them after specific internal processing.

Now you can proceed with artifacts removal. NBT provides several functions to help in this (e.g., an interface for visual inspection of bad channels and noisy epochs, Independent Component Analysis functions for removing periodic artifacts, and different semi-automatic algorithms for facilitating the data cleaning process), but we will not go into details here. However, we would like to emphasize that large-amplitude transient artifacts will influence the temporal structure of the signal and, therefore, it is better to remove them prior to DFA computation (Chen et al., 2002).

### Filter the signal and extract the amplitude envelope

First, we use the function nbt_GetAmplitudeEnvelope to filter the signal using a FIR filter and get the amplitude envelope using the Hilbert transform, *[AmplitudeEnvelope, AmplitudeEnvelopeInfo] = nbt_GetAmplitudeEnvelope(Signal, SignalInfo, hp, lp, filter_order)*. Let us assume that we want to find the DFA in the alpha frequency band (8–13 Hz):


[AmplitudeEnvelope,AmplitudeEnvelopeInfo]
=nbt_GetAmplitudeEnvelope
 (Signal, SignalInfo, 8, 13, 2/8);



Note the last parameter 2/8. This is the filter order (in seconds), which we set such that at least two 8 Hz oscillations cycles are covered by the filter window.

### Perform DFA

The DFA exponents can be then computed using the function nbt_doDFA defined as follow: *[DFAobject,DFA_exp]=nbt_doDFA (Signal, SignalInfo, FitInterval, CalcInterval, DFA_Overlap,DFA_ Plot, ChannelToPlot, res_logbin)*.

The parameters, *FitInterval* and *Calcinterval*, determine the time windows in seconds over which we fit and calculate respectively. The *DFA_overlap* tells how much overlap we want between our windows (in this case 50%, see below). The plotting parameters *DFA_plot* assumes value 1 if you want to visualize the result, otherwise 0; in *ChannelToPlot* you can specify for which channel you want to plot the fluctuation function. The last parameter is the resolution of the logarithmic binning, which by default is 10 per decade.

Now find the DFA exponents and visualize the fluctuation function by typing:


[DFAobject,DFA_exp]=nbt_doDFA
  (AmplitudeEnvelope,AmplitudeEnvelopeInfo,
  [2 25], [0.8 30], 0.5, 1, 1, []);



This instruction will calculate the fluctuation function with 50% overlapping windows from 0.8 to 30 s, and find the DFA exponent by fitting in the interval from 2 to 25 s. The DFA exponent will be stored in *DFA_exp* and *DFA_object* is a structure that stores information such as the fluctuation for each time window and the parameters used to calculate the DFA.

## Insights from the Application of DFA to Neuronal Oscillations

**Figure 7:**
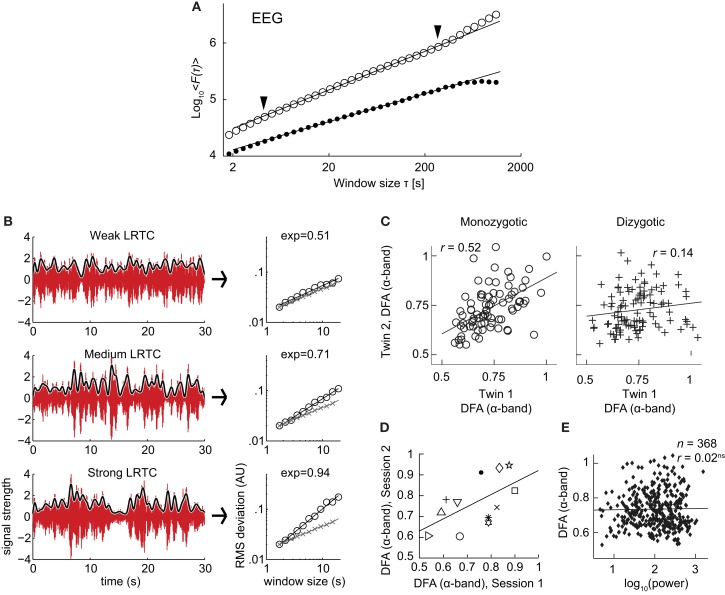
**Results of applying DFA to neuronal oscillations**. **(A)** Robust long-range temporal correlations are observed in the amplitude envelope of human EEG alpha oscillations using the DFA. *Circles*, eyes-closed rest condition; *Dots*, surrogate data (Figure modified from Linkenkaer-Hansen et al., 2001). **(B)** Differences in the scale-free modulation of the amplitude envelope of neuronal oscillations are prominent among individuals and can be quantified using DFA. Here shown for three filtered EEG signals (6–13 Hz) with weak (*top*), medium (*middle*), and strong (*bottom)* LRTC (from channel O2). The gray lines represent the amplitude envelope (low-pass filtered, 1 Hz). DFA fluctuation functions are shown to the right, with signal (*circles)*, and white-noise (*crosses*). The DFA exponent is the slope of the fluctuation function. (Figure modified from Smit et al., 2011). **(C)** Individual differences in long-range temporal correlations in alpha oscillations are to a large extent accounted for by genetic variation, as seen by the difference in correlations of DFA exponents between monozygotic and dizygotic twins (Figure modified from Linkenkaer-Hansen et al., 2007). **(D)** DFA has high test-retest reliability. DFA exponents from the amplitude modulation of alpha oscillations, two sessions with an interval of 6–28 days, symbols indicates different subjects (Figure modified from Nikulin and Brismar, 2004). **(E)** The DFA exponent is independent of oscillation power. Data were recorded using EEG on 368 subjects during a 3 min eyes-closed rest session (Figure modified from Linkenkaer-Hansen et al., 2007).

The discovery of LRTC in the amplitude envelope of ongoing oscillations, was based on 10 subjects recorded with EEG and MEG for 20 min during eyes-closed and eyes-open rest (Linkenkaer-Hansen et al., 2001). In both conditions, amplitude envelopes of alpha and beta oscillations exhibited power-law scaling behavior on time-scales of 5–300 s with DFA exponents significantly higher than for band-pass filtered white-noise (Figure 7A). These results were further validated by showing 1/*f* power spectra and a power-law decay in the auto-correlation function.

The robustness of LRTC in ongoing oscillations has been confirmed in several follow-up studies, albeit often based on shorter experiments and scaling analysis in the range of about 1–25 s (Linkenkaer-Hansen et al., 2007; Monto et al., 2007; Berthouze et al., 2010; Smit et al., 2011; Figure 7B). The power-law scaling behavior in the theta band is reported less often (Smit et al., 2011), and to our knowledge LRTC in the delta band have only been investigated in subdural EEG (Monto et al., 2007). LRTC have also not been reported often in the gamma band due to the low SNR obtained from EEG/MEG recordings in this band. Invasive recordings in non-human primates, however, have reported 1/*f* spectra for the amplitude modulation in both low and high gamma bands (Leopold et al., 2003). Recordings from the subthalamic nucleus in Parkinson patients even show prominent LRTC in the very high-frequency gamma range (>200 Hz), especially when treated with the dopamine-precursor drug Levodopa (Hohlefeld et al., 2012).

To gain validity for LRTC it has been shown that LRTC have a link to the underlying genetics of the subject. This link was provided in (Linkenkaer-Hansen et al., 2007) where the scaling of eyes-closed rest EEG from monozygotic and dizygotic twin subjects (*n* = 368) showed that ∼60% of the variance of DFA exponents in the alpha- and beta-frequency bands is attributable to genetic factors (Figure 7C). This was an important result as it clearly showed that the non-random patterns of fluctuations in the ongoing oscillations are governed by low-level biological factors as opposed to uncontrolled experimental variables during the recording sessions. The finding also provides an explanation of the significant test-retest reliability of DFA exponents (Figure 7D; Nikulin and Brismar, 2004).

Several studies have reported that DFA exponents of neuronal oscillations are independent of oscillation power for a given frequency band, both when the oscillations are recorded with subdural EEG (Monto et al., 2007) and scalp EEG (Linkenkaer-Hansen et al., 2007; Smit et al., 2011; Figure 7E). These results together indicate that the DFA can be used as a robust measure of oscillatory dynamics, which captures different features of brain activity than those seen in classical analysis such as power in a frequency band.

### DFA as a biomarker of neurophysiological disorder

**Figure 8:**
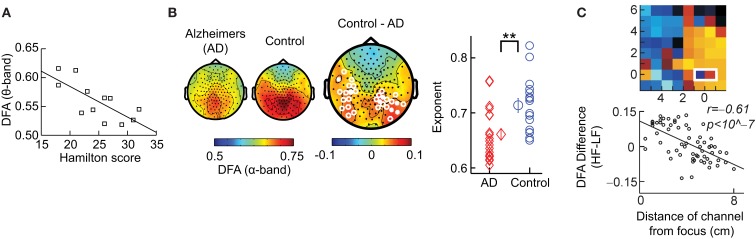
**DFA is a promising biomarker for pre-clinical studies**. **(A)** DFA exponents of theta oscillations in left sensorimotor region correlate with the severity of depression based on the Hamilton score. Data recorded from 12 depressed patients with MEG, during an eyes-closed rest session of 16 min. (Figure modified from Linkenkaer-Hansen et al., 2005). **(B)** DFA of alpha oscillations shows a significant decrease in the parietal area in patients with Alzheimer’s disease than in controls. MEG was recorded during 4 min of eyes-closed rest and the DFA exponent estimated in the time range of 1–25 s. (*Right*) Individual-subject DFA exponents averaged across significant channels are shown for the patients diagnosed with early stage Alzheimer’s disease (*n* = 19) and the age-matched control subjects (*n* = 16; Figure modified from Montez et al., 2009). **(C)** Difference in the DFA exponent of high frequencies (beta band) and low frequencies (alpha band) indicates the location of the epileptic focus (*white box*). Data recorded from an epileptic subject using subdural EEG during seizure-free activity (modified from Monto et al., 2007 by permission of Oxford University Press).

We have so far discussed the results of applying DFA to healthy subjects; however, some of the most exciting results have come from pre-clinical studies, which indicate possible functional roles for LRTC. For example, a breakdown of LRTC in the amplitude fluctuations of resting-state theta oscillations detected in the left sensorimotor region was reported for patients with major depressive disorder (Linkenkaer-Hansen et al., 2005). Interestingly, the severity of depression, as measured by the Hamilton depression rating scale, inversely correlated with the DFA exponent of the patients (Figure 8A). Reduction in the LRTC of oscillations has also been reported in the alpha band in the parietal region in patients with Alzheimer’s disease (Montez et al., 2009; Figure 8B). Furthermore, reduction in the alpha and beta bands in the centro-parietal and fronto-central areas has also been reported for patients with schizophrenia (Nikulin et al., 2012).

Interestingly, it seems as though it is not only a loss of LRTC that correlates with disorders, but also elevated levels of LRTC. A study (Monto et al., 2007) looked at different scales of neuronal activity by using subdural EEG to record the areas surrounding an epileptic focus in five patients during ongoing seizure-free activity. They discovered that the LRTC are abnormally strong near the seizure onset zone (Figure 8C). Further, it was shown that administration of the benzodiazepine lorazepam to the patients, leads to decreased DFA exponents in the epileptic focus, suggesting that the pharmacological normalization of seizure activity brings with it also a normalization of LRTC. Interestingly, however, DFA exponents were observed to increase in the seizure-free surrounding areas, which may correspond to the increase in LRTC observed *in vitro* after application of Zolpidem, which is also a GABAergic modulator (Poil et al., 2011).

Overall these studies seem to indicate that there is an optimal level of temporal structure of oscillations and any deviation from this can result in a significant loss of function (Poil et al., 2012). Importantly, whereas early studies have estimated the DFA exponent from the scaling of the fluctuation function across almost two orders of magnitude in time (Linkenkaer-Hansen et al., 2001, 2004; Parish et al., 2004; Monto et al., 2007), most reports have used one decade of fitting range and found the DFA a very useful biomarker to study neuronal dynamics in health and disease.

## Outlook

In the last 10 years there has been rapid progress in the field of LRTC analysis of neuronal signals (Linkenkaer-Hansen et al., 2001; Parish et al., 2004; Stead et al., 2005; Monto et al., 2007). However, there are still many fundamental issues that need to be addressed, thus presenting many exciting opportunities for applying LRTC methodology to studies of normal and pathologic brain functioning.

It has for a long time been recognized that the brain functions at different time-scales, ranging from a few tens of milliseconds required for the perception of stimuli, to tens of seconds spent on different cognitive operations (Axmacher et al., 2006; Buzsáki, 2006; Cassenaer and Laurent, 2007; Lisman, 2010). Yet, rarely were neuronal dynamics studied with approaches incorporating different time-scales in order to better understand integrative brain mechanisms. In this sense LRTC represent a unique approach describing in a succinct way how neuronal activity unfolds in time taking into account different time-scales. Given that neuronal signals are often non-stationary, DFA has been proven to be a reliable method for capturing LRTC. The DFA method can be successfully applied to both resting-state and task-dependent recordings. It can also be used for quantifying brain activity during different tasks, such as mental counting, visual and motor imagery, or even during presentation of different stimuli. Here the neuronal reactivity caused by the stimuli is usually transient in the order of hundreds of milliseconds and as such can easily be ruled out as the source for modulation of neuronal dynamics on the scale of tens of seconds (Linkenkaer-Hansen et al., 2004), the latter rather being related to the attentional or vigilance states. Recently DFA has been adapted to allow detection of time-varying scaling exponents (Berthouze and Farmer, 2012), which could prove useful in data where brain-state changes could be expected to produce different scaling, e.g., at the onset of sleep (Kim et al., 2009) or in acute response to drugs (Monto et al., 2007; Hohlefeld et al., 2012).

In (Monto et al., 2008) it was shown that there are infraslow oscillations with a frequency of 0.01–0.1 Hz that predict human behavioral performance and were correlated with the amplitude of the classical frequency bands (alpha, beta, gamma, etc.). However, it is yet to be determined whether the amplitude modulation of the classical frequency band oscillations are the cause of infraslow oscillations, which is theoretically plausible, because these oscillations often have non-zero mean (Nikulin et al., 2007). Alternatively, a mechanism that is not directly related to the neuronal oscillations could produce excitability changes in the cortex, which would be reflected in infraslow oscillations and modulate the amplitude of all the other oscillations.

One of the main explanations for the presence of LRTC in neuronal oscillations has been the hypothesis of a brain being in a critical-state (Bak, 1996; Linkenkaer-Hansen et al., 2001; Kello et al., 2010). Criticality in neuronal networks has been related to optimal information processing using computational models (Kinouchi and Copelli, 2006). At the level of neuronal populations, criticality is reflected in scale-free distributions of local field potential propagations, so-called neuronal avalanches, and these have been observed both *in vitro* (Beggs and Plenz, 2003) and *in vivo* (Petermann et al., 2009). Importantly, it was recently shown in computational models of neuronal oscillations that LRTC emerges only when networks produce critical neuronal avalanches and this occurs when excitatory and inhibitory connectivities are balanced (Poil et al., 2012). Thus, it is likely that LRTC reflect critical-state dynamics of neuronal networks, but more work is needed to explain how variation in DFA exponents in different frequency bands and anatomical regions relate to neuronal avalanches, criticality, and computation.

## Conflict of Interest Statement

The authors declare that the research was conducted in the absence of any commercial or financial relationships that could be construed as a potential conflict of interest.

## Footnotes

^1^http://www.physionet.org
